# *Burkholderia pseudomallei* PenI β-lactamase and variants are potently inhibited by taniborbactam

**DOI:** 10.1128/aac.00787-25

**Published:** 2025-09-12

**Authors:** Maria F. Mojica, Scott A. Becka, Mitchell Edwards, Cullen Myers, Kyoko Uehara, Tsuyoshi Uehara, Tyuji Hoshino, Elise T. Zeiser, Cassandra L. Chatwin, David A. Six, Robert A. Bonomo, Krisztina M. Papp-Wallace, Michiyoshi Nukaga

**Affiliations:** 1 Department of Molecular Biology and Microbiology, Case Western Reserve University School of Medicine 12304, Cleveland, Ohio, USA; 2 Research Service, Veterans Affairs Northeast Ohio Healthcare System 465630 , Cleveland, Ohio, USA; 3 CASE-VA Center for Antimicrobial Resistance and Epidemiology, Cleveland, Ohio, USA; 4 Venatorx Pharmaceuticals Inc, Malvern, Pennsylvania, USA; 5 Graduate School of Pharmaceutical Sciences, Chiba University 12737, Chiba, Japan; 6 Geriatric Research, Education and Clinical Center, Louis Stokes Cleveland VA Medical Center 20083, Cleveland, Ohio, USA; 7 Department of Medicine, Case Western Reserve University School of Medicine 12304, Cleveland, Ohio, USA; 8 Department of Biochemistry, Case Western Reserve University School of Medicine 12304, Cleveland, Ohio, USA; 9 Department of Pharmacology, Case Western Reserve University School of Medicine 12304, Cleveland, Ohio, USA; 10 Department of Proteomics and Bioinformatics, Case Western Reserve University School of Medicine 12304, Cleveland, Ohio, USA; 11 Department of Pharmaceutical Sciences, Josai International University 12840, Togane City, Chiba, Japan; University of Fribourg, Fribourg, Switzerland

**Keywords:** *Burkholderia pseudomallei*, cefepime, ceftazidime, melioidosis, PenI, resistance, taniborbactam

## Abstract

*Burkholderia pseudomallei* is a Gram-negative pathogen that causes melioidosis, a severe and often fatal disease. Due to its recognized potential as a bioterrorism agent, it is critical that appropriate antimicrobial agents are available for post-exposure prophylaxis and treatment of melioidosis. Cefepime–taniborbactam is a novel β-lactam–β-lactamase inhibitor combination in clinical development, with promising activity against Gram-negative bacteria producing class A, B, C, and/or D β-lactamases. Herein, we demonstrate the inhibitory activity of taniborbactam against PenI, the class A β-lactamase produced by *B. pseudomallei*. Isogenic *Escherichia coli* strains producing PenI and its ceftazidime-resistance–conferring variants (C69Y and P167S) showed ceftazidime minimum inhibitory concentration (MIC) of 64 mg/L for the strain producing PenI and 1,024 mg/L for the strains producing the variants, whereas cefepime MIC was 128–256 mg/L for these three strains. While the addition of avibactam decreased ceftazidime MIC to 1 mg/L for PenI and 8–16 mg/L for the variants, the addition of taniborbactam decreased cefepime MIC to ≤0.5 mg/L for PenI and the variants. Similarly, an 8-fold reduction of the cefepime MIC upon addition of taniborbactam was observed in an avirulent *B. pseudomallei* strain. Furthermore, taniborbactam inhibited PenI and its C69Y variant with an apparent *K*_i_ of 0.11 and 3.1 µM, respectively. Lastly, co-crystallography and molecular dynamics simulations showed that taniborbactam induced the formation of a disulfide bond between Cys77 and Cys123, which destabilizes the deacylation water and strengthens the taniborbactam–PenI complex. These results support the development of cefepime–taniborbactam as a promising agent for the treatment of infections by *B. pseudomallei*.

## INTRODUCTION

*Burkholderia* spp. is a complex set of Gram-negative bacteria found in a wide range of ecological niches. *Burkholderia* spp. can be transferred from a natural reservoir such as water and soil to humans and animals by direct contact or through contaminated secondary reservoirs (e.g., disinfectants, saline, mouthwash, and water). Major *Burkholderia* spp. human pathogens include the *B. pseudomallei* complex, *B. cepacia* complex (Bcc), and *B. gladioli* (1, 2).

*B. pseudomallei* is a facultative intracellular pathogen and the causative agent of the severe and often fatal disease melioidosis (3). *B. pseudomallei* can infect humans through inhaling contaminated dust, thereby posing as a potential biothreat pathogen that could be used in biological warfare and bioterrorism (Tier 1 Select Agent) (4). A striking feature of melioidosis is a high mortality rate (up to 60% of treated patients succumb to pneumonia) (5). Recently, it was reported that *B. pseudomallei* was isolated from the soil of a Mississippi Gulf Coast County and was the source of infection of three patients between 2020 and 2023 (6). An additional case of melioidosis was reported from a resident of Texas with no travel history (7). From these reports, the US National Institute of Allergy and Infectious Diseases (NIAID), in conjunction with the US Department of Homeland Security and the Centers for Disease Control and Prevention (CDC), has defined *B. pseudomallei* as a Category B Priority pathogen in the Emerging Infectious Diseases/Pathogens priority list (8).

*B. pseudomallei* is naturally resistant to a variety of antibiotics, including cephalosporins, penicillins, macrolides, rifamycins, aminoglycosides, and polymyxins (9). In *B. pseudomallei*, β-lactam resistance is directly correlated with the presence of a chromosomally encoded class A β-lactamase, PenI (10) (formerly known as BPS-1 (11) or PenA (12), which was first described in 1987 (13). PenI was found to be an extended-spectrum β-lactamase (ESBL) (11–14). In addition, *B. pseudomallei* also carries a chromosomally encoded class D OXA β-lactamase (15–17). The current recommended agent of the initial intensive therapy for *B. pseudomallei* infection is an intravenously administered β-lactam, ceftazidime (CAZ), or meropenem (18), neither of which is inactivated effectively by these β-lactamases. However, the development of PenI-mediated CAZ resistance during treatment is well documented (19–21). The mechanisms of PenI-dependent CAZ resistance include upregulation of PenI expression due to promoter mutation (19, 22–24), gene duplication and amplification (25), and variants of PenI with presumably enhanced activity against CAZ (12, 19, 24, 26, 27). High-level CAZ-resistant isolates (MIC ≥ 256 µg/mL) have been shown to express a PenI variant that has a cysteine to tyrosine (C69Y) substitution at amino acid position 69, and the PenI variant is sufficient to confer high-level CAZ resistance when recombinantly expressed in *E. coli* (19). Another variant, PenI P167S, has also been shown to elevate CAZ MIC (12, 21).

To overcome resistance mediated by β-lactamases, β-lactamase inhibitors (e.g., boronates and diazabicyclooctanes [DBOs]) are often used in combination with a β-lactam (28). The two novel β-lactam/DBO combinations, ceftazidime–avibactam and sulbactam–durlobactam, show *in vitro* antibacterial activity against strains of *B. pseudomallei* (29, 30). Overall, β-lactam resistance rates in *B. pseudomallei* remain low (21); however, selection of resistant strains during treatment has been reported (12, 20, 27, 31–33). Thus, due to the potential use of *B. pseudomallei* for bioterrorism, it is critical that appropriate antimicrobials for treatment and post-exposure prophylaxis are available.

**Fig 1:**
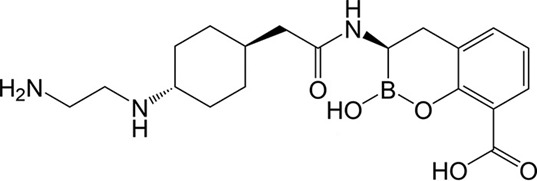
Chemical structure of taniborbactam (VNRX-5133).

In this regard, we sought to explore the potential role of the novel combination of cefepime (FEP)–taniborbactam (VNRX-5133), being developed by Venatorx Pharmaceuticals (34), as an alternative treatment of *B. pseudomallei* infections. Early *in vitro* (35) and *in vivo* (36) research showed that FEP is not as active as CAZ against *B. pseudomallei*. In fact, a recent publication demonstrated that 41% of the *B. pseudomallei* isolates are resistant to this antibiotic (36). Nevertheless, FEP has been used in combination with trimethoprim–sulfamethoxazole for the treatment of melioidosis (37). Taniborbactam is a boronic acid β-lactamase inhibitor with a broad spectrum of activity that covers not only class A, C, and D serine β-lactamases but also many class B metallo-β-lactamases, including NDM (except NDM-9 and NDM-30) and VIM (except VIM-83; Fig. 1) (38). The cefepime–taniborbactam combination demonstrated efficacy in various murine infection models caused by cephalosporin-resistant *Klebsiella pneumoniae* and carbapenem-resistant *Enterobacterales*, including metallo-β-lactamase producers (39–43). Furthermore, a phase 3 clinical trial comparing cefepime–taniborbactam to meropenem for the treatment of complicated urinary tract infections and acute pyelonephritis demonstrated superior efficacy of cefepime–taniborbactam to meropenem with a safety profile similar to that of meropenem. The most common pathogens identified were *Enterobacterales* species (95.6%), mainly *Escherichia coli* (69.0%), *K. pneumoniae* (13.8%), and *Proteus mirabilis* (4.6%). *Pseudomonas aeruginosa* was detected in 4.1% of the cases. (44)

In this study, we conducted a comprehensive biochemical characterization of the interactions between PenI and taniborbactam, including co-crystallization and molecular dynamics simulation studies, to discern the structural interactions that allow for a potent inhibition.

## RESULTS

### Antimicrobial susceptibility testing using isogenic *E. coli* producing PenI and its C69Y and P167S variants demonstrates potent activity of cefepime–taniborbactam

To evaluate the ability of taniborbactam to inhibit wild-type PenI and its C69Y and P167S variants, MIC values were determined in isogenic strains based on the *E. coli* K-12 DH10B strain. This strain had minimal background β-lactamase activity against CAZ and FEP originating from low-level expression of the EC-1 class C cephalosporinase (AmpC). As shown in Table 1, heterologous expression of wild-type *bla*_PenI_ from a high-copy-number plasmid increased the MIC values for both CAZ and FEP >100-fold. The addition of avibactam (AVI) to CAZ reduced the MIC value to within 2-fold of the vector control, indicating that AVI potently blocks the β-lactamase activity of PenI. Similarly, the addition of taniborbactam to FEP reduced the MIC value 2,000-fold, reaching the background FEP resistance observed for the vector control and demonstrating that taniborbactam is a potent inhibitor of PenI. Expression of the CAZ-resistant PenI variants (C69Y or P167S) led to a significantly higher CAZ MIC value (≥1024 µg/mL) compared to the isogenic strain expressing wild-type PenI (64 µg/mL). For these variants, the addition of AVI reduced the CAZ MIC to 8–16 µg/mL but failed to reach levels seen for the vector control (0.5 µg/mL). In contrast, the CAZ-resistant PenI variants did not exhibit any significant change in the FEP MIC value compared to wild-type PenI. Moreover, for each PenI variant, the addition of taniborbactam reduced the FEP MIC to within 4-fold of FEP alone in the vector control (0.12 µg/mL).

**TABLE 1:** MIC and β-lactamase activity of parent and PenI expressing isogenic *E. coli* strains

*E. coli* DH10B carrying	β-lactamase^*a*^	Minimum inhibitory concentration (µg/mL)				Relative β-lactamase activity ± SD^*b*^
		CAZ	CAZ + AVI^*c*^	FEP	FEP + TAN^*c*^	
pTU646	Vector control	0.5	0.5	0.25	0.12	1.00
pTU689	PenI	64	1	256	0.12	497 ± 73
pTU690	PenI C69Y	1024	8	128	0.5	0.82 ± 0.06
pTU777	PenI P167S	1024	16	128	0.25	45.10 ± 4.70

^*a*^ Because PenI is secreted by the twin arginine translocation (TAT) system (45), the mature protein of PenI was fused to Tss, a TAT signal sequence in *E. coli *(46) for proper secretion of folded PenI protein into the periplasm. The vector control plasmid pTU646 expressed only the TorA signal sequence.

^*b*^ Relative values of β-lactamase activity (substrate: nitrocefin) in cell lysates, considering one as the basal activity of the reference strain DH10B/pTU646 (7.5 ± 1.2 nmol of nitrocefin hydrolyzed/min/mg of protein). SD, standard deviation.

^*c*^ AVI and TAN were fixed at 4 µg/mL; AVI, avibactam; CAZ, ceftazidime; FEP, cefepime; TAN, taniborbactam; Modal MIC shown from three replicates.

Notably, a similarly powerful effect of the FEP–taniborbactam combination was observed against an avirulent *B. pseudomallei* isolate, Bp82 (47). For this isolate, the FEP MIC value of 16 µg/mL dropped to 2 µg/mL upon the addition of taniborbactam. This encouraging result warrants further evaluation of cefepime–taniborbactam against clinical *B. pseudomallei* isolates.

### Substrate preferences of PenI and its C69Y variant

**Fig 2:**
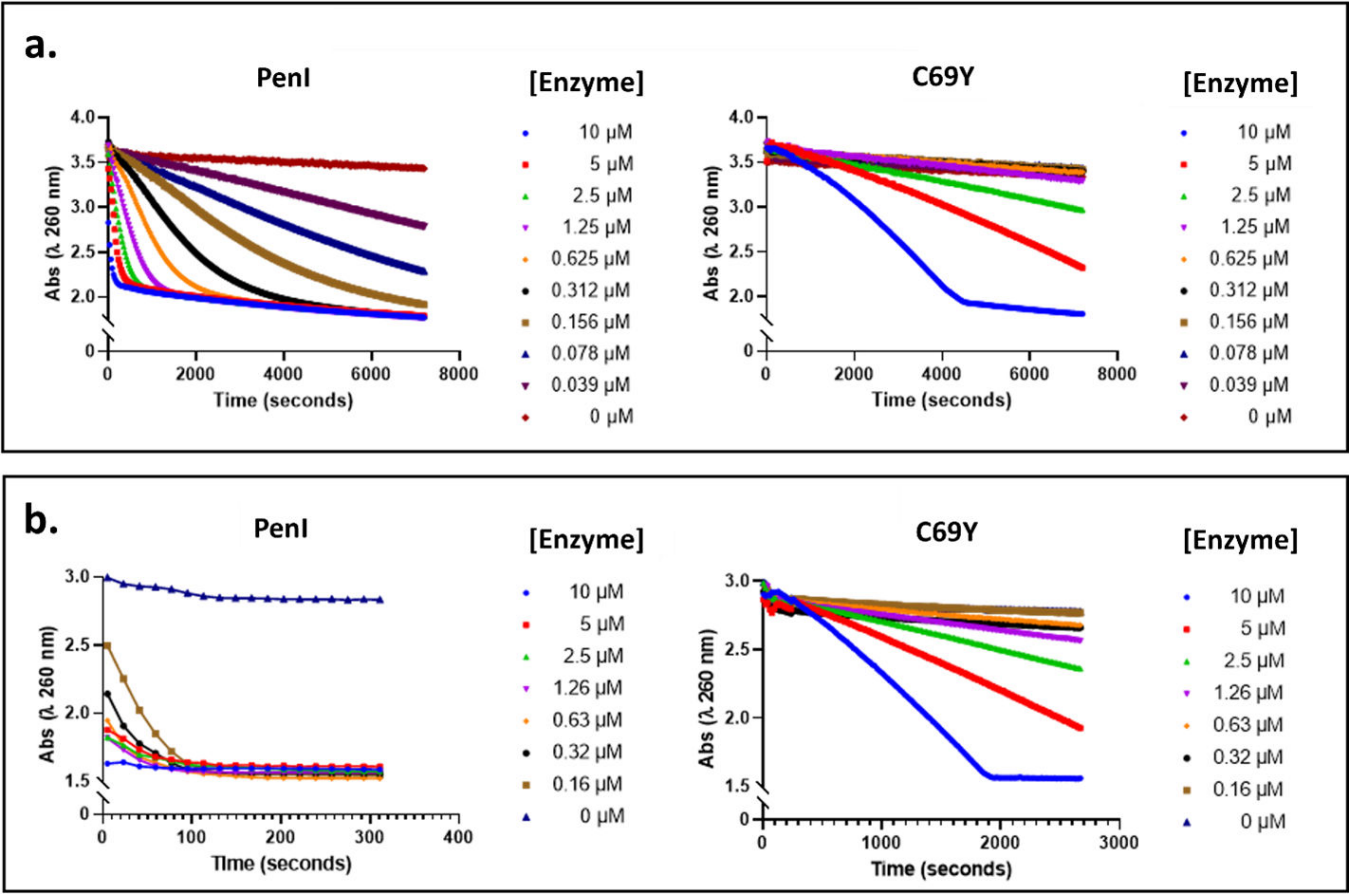
Hydrolysis of CAZ and FEP by PenI and the C69Y variant. CAZ (**a**) and FEP (**b**) hydrolysis were monitored continuously by the reduction in absorbance at 260 nm. At equivalent enzyme concentrations, CAZ and FEP hydrolysis occurred at significantly slower rates for the C69Y variant than for PenI.

PenI and the C69Y variant were purified for *in vitro* experiments to examine the biochemical basis for increased CAZ and FEP resistance conferred by this variant relative to the wild-type enzyme. Quantitative immunoblotting experiments have shown that the C69Y variant (as well as the P167S variant) is expressed at levels compared to the wild-type enzyme in *B. pseudomallei* (48). Surprisingly, however, CAZ or FEP turnover by the C69Y variant was significantly slower than by the wild-type enzyme (Fig. 2). Michaelis–Menten parameters (*K*_M_, *k*_cat_, *k*_cat_/*K*_M_) determined for PenI hydrolysis of CAZ and FEP indicated that FEP, as suggested by the MIC data, was a better PenI substrate, with a *K*_M_ of 178 µM compared to a CAZ *K*_M_ > 500 µM (Table 2). FEP was also more efficiently hydrolyzed than nitrocefin (*k*_cat_/*K*_M_ of 0.28 s^−1^µM^−1^ for FEP vs. 0.13 s^−1^µM^−1^ for nitrocefin) (Table 2). The significantly diminished hydrolysis of CAZ or FEP by the C69Y variant precluded the determination of kinetic parameters for hydrolysis of these substrates. Thus, only the hydrolysis of nitrocefin was characterized, revealing a ~50-fold reduction in *k*_cat_ accompanied by a ~4-fold reduction in the *K*_M_ that resulted in an order of magnitude reduction in the catalytic efficiency (*k*_cat_/*K*_M_). This outcome was reflected by the β-lactamase activity in cell lysates extracted from the isogenic *E. coli* strains, measured using nitrocefin (Table 1). The lysate from *E. coli* cells overproducing wild-type PenI was 600-fold more active than that from cells overproducing the C69Y variant, consistent with the biochemical data using the purified proteins. Notably, the lysate from cells overexpressing the P167S variant also showed 11-fold lower activity compared to that from cells expressing wild-type PenI.

**TABLE 2:** Kinetic parameters for hydrolysis of β-lactam substrates by PenI and the C69Y variant^*a*^

β-lactam substrate	PenI			PenI C69Y		
	*K*_M_ (µM)	*k*_cat_ (sec^−1^)	*k*_cat_/*K*_M_ (sec^−1^µM^−1^)	*K*_M_ (µM)	*k*_cat_ (sec^−1^)	*k*_cat_/*K*_M_ (sec^−1^µM^−1^)
Nitrocefin	44.3 ± 7.4	5.8 ± 0.4	0.131 ± 0.016	12.2 ± 2.2	0.120 ± 0.001	0.010 ± 0.002
Cefepime	178 ± 15	50.0 ± 1.4	0.28 ± 0.02	ND^*c*^	ND	ND
Ceftazidime	>500			ND	ND	ND
Meropenem	NHD	NHD	NHD	NHD^*b*^	NHD	NHD

^*a*^ Empty cells indicate the rate of CAZ hydrolysis was not saturable at the highest concentration tested so *k*_cat_ and *k_c_*_at_/*K*_M_ could not be accurately determined.

^*b*^ NHD, no hydrolysis detected.

^*c*^ ND, not determined.

**Fig 3:**
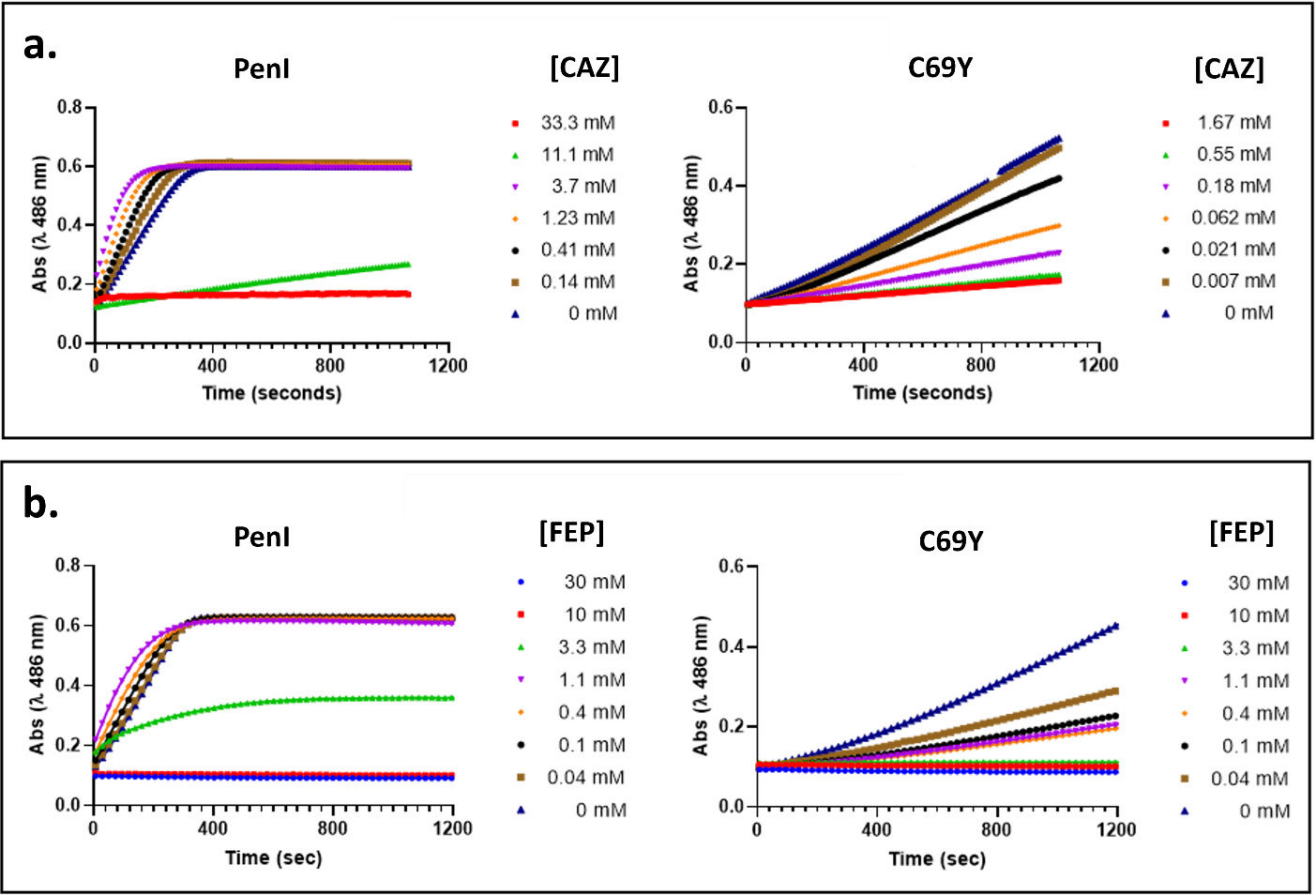
Inhibition of PenI and the C69Y variant Nitrocefin hydrolysis by CAZ and FEP. Nitrocefin hydrolysis was monitored continuously by absorbance at 486 nm. The presence of increasing concentrations of CAZ (**a**) or FEP **(b**) inhibited nitrocefin hydrolysis by PenI and the C69Y variant, consistent with competition for the β-lactamase active site.

The weaker hydrolytic activity observed for the C69Y variant seemingly contradicts MIC data showing a substantially elevated CAZ MIC and no impact to the FEP MIC in a C69Y variant expression background. To further investigate this discrepancy, relative affinities of CAZ and FEP for the C69Y variant and wild-type PenI were assessed in competition assays using nitrocefin as a reporter substrate. With both CAZ and FEP, the inhibition of PenI-mediated nitrocefin hydrolysis became evident at >1 mM (Fig. 3), whereas nitrocefin hydrolysis by the C69Y variant was inhibited at markedly lower concentrations (<0.1 mM), suggesting a higher affinity of these antibiotics for the variant (Fig. 3).

### Taniborbactam is a potent inhibitor of PenI and its C69Y variant

The inhibition of wild-type PenI was tested with avibactam (a DBO), taniborbactam and vaborbactam (cyclic boronates), and clavulanic acid and tazobactam (mechanism-based β-lactam inhibitors). Consistent with the MIC data, taniborbactam exhibited robust inhibition of PenI, demonstrating superior inhibitory properties (IC_50_, *k*_2_/*K*, off-rate/half-life) compared to avibactam or vaborbactam (Table 3). To assess inhibition of the C69Y variant relative to wild-type PenI, apparent *K*_i_ values were determined for taniborbactam and comparator BLIs, and both avibactam and taniborbactam were found to inhibit the C69Y variant with reduced potencies relative to wild-type PenI. Importantly, taniborbactam was 15-fold more potent than avibactam against PenI and 2-fold more potent against the C69Y variant (Table 4). In contrast, vaborbactam only weakly inhibited PenI and showed no apparent inhibition of the C69Y variant at the highest concentration tested.

**TABLE 3:** Potency and inhibition kinetics for β-lactamase inhibitors with PenI^*a*^

β-lactamase inhibitor	IC_50_ (nM)	*k*_2_/*K* (10^5^ × M^−1^s^−1^)	*k*_off_ (10^−4^ × s^−1^)	t_1/2_ (min)
Avibactam	127 ± 7	0.41 ± 0.02	15.6 ± 1.1	7.4 ± 0.5
Taniborbactam	5.2 ± 0.9	4.59 ± 0.19	4.3 ± 0.9	27.4 ± 5.5
Vaborbactam	98 ± 26	0.061 ± 0.002	9.0 ± 0.3	12.8 ± 0.5
Clavulanic acid	249 ± 67	nt	nt	nt
Tazobactam	88.5 ± 2.3	nt	nt	nt

^*a*^ nt, not tested.

**TABLE 4:** Apparent *K*_i_ of β-lactamase Inhibitors with PenI and the C69Y Variant

β-lactamase inhibitor	Apparent *K*_i_ (µM)	
	PenI	C69Y
Avibactam	1.64 ± 0.15	6.7 ± 0.2
Taniborbactam	0.11 ± 0.04	3.10 ± 0.03
Vaborbactam	>10	> 100

### Crystallography reveals key interactions for the potent inhibition of PenI by taniborbactam

**Fig 4:**
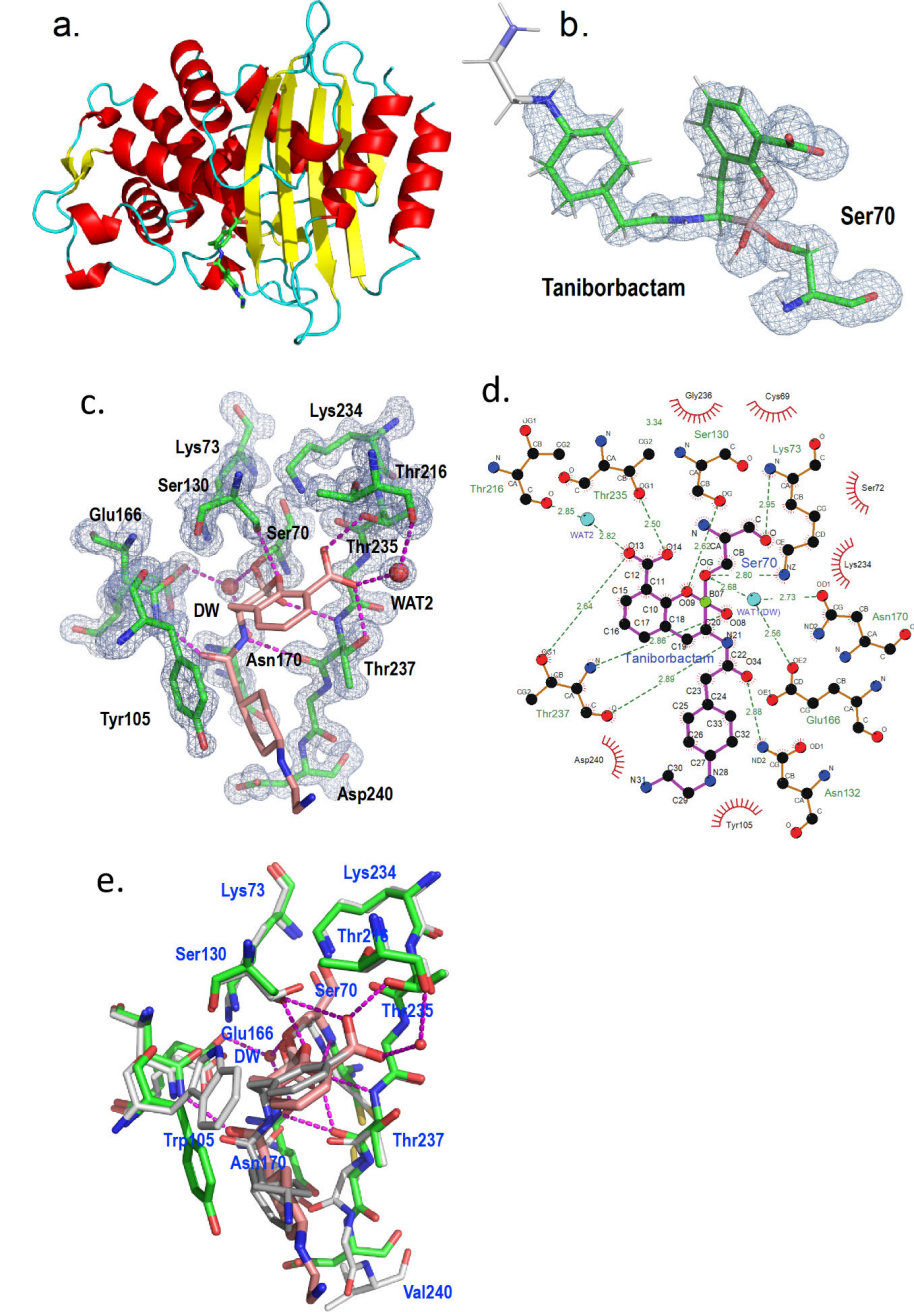
PenI–taniborbactam crystal structure. (**a**) Ribbon representation of the PenI–taniborbactam complex. (**b**) Polder map (omit map) calculated by PHENIX at 3.5 σ. Taniborbactam bound to Ser70 is shown in green. The white-colored portion at the end of taniborbactam has an occupancy of 0.3, because the electron density was not observed in the 2.5 σ polder map. (**c**) 2Fo-Fc electron density map (1.5 σ) around the active site of PenI–taniborbactam complex. Amino acids are shown in green and taniborbactam in pink. (**d**) A Ligplot drawing of PenI–taniborbactam complex active site. (**e**) Superposition of PenI–taniborbactam (green) and the KPC-2:taniborbactam (49) complex (light gray, PDB: 6TD1). Abbreviations: DW (WAT1), deacylation water; WAT2, water forming hydrogen-bonding interactions.

**Fig 5:**
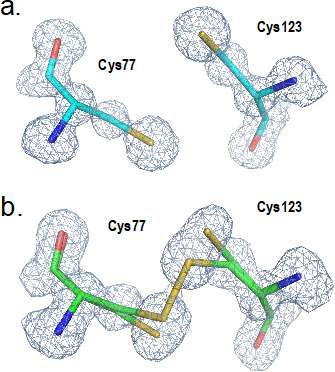
Polder map (omit map, contour level at 3.0 σ) of Cys77 and Cys123 region. (**a**) Apo-PenI and (**b**) PenI–taniborbactam complex. In the complex, both the disulfide bond and free thiols were modeled.

To understand the structural basis of the inhibition of PenI by taniborbactam, the crystal structure of the complex was determined. The PenI–taniborbactam complex structure was solved in space group *P*2_1_ at 1.25 Å resolution. With anisotropic B-factor and riding hydrogen for the PenI protein model, *R*/*R*_free_ values were 0.125/0.151 (Table S1; Fig. 4a). The tail end of taniborbactam could not be resolved, as poor electron density was observed for the 2*F*_o_-*F*_c_ map at 1.0 σ and the polder map (omit map) at 2.5 σ in this region; thus, the atom occupancies were set to 0.3 (Fig. 4b). The high resolution of the active site allowed detection of the boron of taniborbactam bound to Ser70, as well as other direct hydrogen-bonding interactions of taniborbactam with residues Ser130, Asn132, Thr235, and Thr237 and water-bridged hydrogen-bonding interactions with Glu166 and Thr216 (Fig. 4c and d). Therefore, the overall binding mode was like that observed in the KPC-2 structure in complex with taniborbactam (49), with some differences (Fig. 4e). For instance, the orientation of Tyr105 in PenI was different from that of Trp105 in the KPC-2/taniborbactam complex. In PenI, Tyr105 limited the freedom of taniborbactam to have alternate conformations. Also, in the PenI–taniborbactam complex, a disulfide bond between Cys77 and Cys123 as well as free thiols was observed with the ratio between the two at approximately 1:1 (Fig. 5). Of note, in the apo-PenI structure, only the free thiols were observed (14); thus, a disulfide bond appears to have formed upon complexing with taniborbactam. However, we cannot exclude the possibility that the procedure of protein preparation may affect the formation of a disulfide bond because different protein stocks were used to solve the apo-PenI structure (14) and the PenI–taniborbactam structure (this study).

### Molecular dynamics (MD) simulation reveals that the presence of the Cys77–Cys133 disulfide bond might affect the stability of the deacylation water

**Fig 6:**
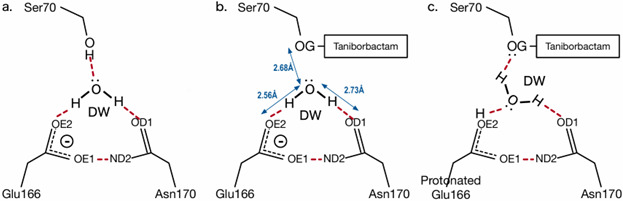
Hydrogen bonding network around the deacylation water used in the six MD simulations. (**a**) This configuration was used for PenI-Ox and PenI-Red simulations. (**b**) This configuration was used for PenI-Ox-TAN and PenI-Red-TAN simulations. The bond length values were obtained from the PenI–taniborbactam crystal structure. (**C**) This configuration with Glu166 protonated was used for PenI-Ox-TAN-166 and PenI-Red-TAN-166 simulations.

The hydrogen bond network around the deacylation water of class A β-lactamase has been elucidated through X-ray crystallography. In the apoenzyme (Fig. 6a), Ser70 (OG) acts as a hydrogen donor, while the deacylation water serves as a hydrogen acceptor, forming bonds with ionic Glu166 (OE1) and Asn170 (NE) (50). In the acyl intermediate, hydrogen bonds are formed by ionic Glu166 and Asn170, enhancing the nucleophilicity of the deacylation water and allowing it to attack the carbonyl carbon of the acyl intermediate. Of note, in the complex of inactivated class A β-lactamase SHV-1 with meropenem, protonated Glu166 acts as a proton donor, altering the orientation of the lone pair of electrons (51). Thus, for the PenI–taniborbactam complex, we considered both ionic, deprotonated Glu166 (Fig. 6b) and neutral, protonated Glu166 (Fig. 6c) in our initial structure for the molecular dynamic calculations. To investigate whether and how the formation of the Cys77–Cys123 disulfide bond affects the total structure and movement of the main chain amino acids, a 200 ns simulation was conducted with apo-PenI (Fig. 6a) with (PenI-Ox) and without the disulfide bond (PenI-Red); PenI–taniborbactam complex (Fig. 6b) with (PenI-Ox-TAN) and without the disulfide bond (PenI-Red-TAN); and PenI with Glu166 protonated (Fig. 6c) which was used for both the PenI–taniborbactam complex with (Pen-Ox-TAN-166) or without the disulfide bond (PenI-Red-TAN-166). RMSD values of the apo-PenI structures and PenI–taniborbactam complexes for main chain atoms from the initial structure were stable regardless of the presence or absence of the disulfide bond (Fig. S1a and b). Likewise, the B-factor profiles were very similar for the six MD simulations (Fig. S1c and d). This observation suggests that the total structure and the movement of main chain amino acids are not affected by the disulfide bond formation, taniborbactam binding, nor the state of Glu166.

**Fig 7:**
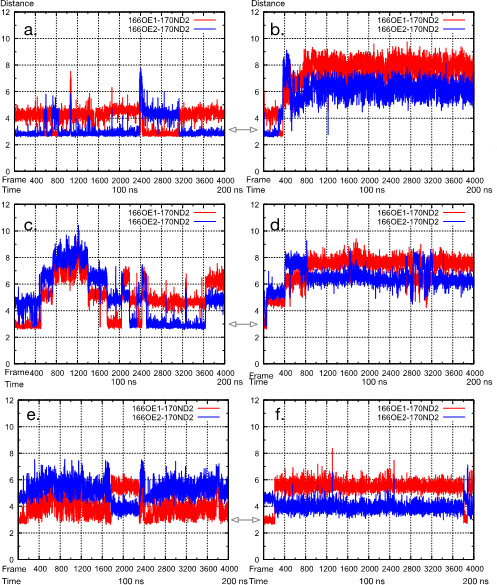
Observed changes in distance between Glu166:OD1,2-Asn170:ND2 during the 200 ns simulation. (**a**) apo-PenI without a disulfide (PenI-Red), (**b**) apo-PenI with a disulfide (PenI-OX), (**c**) PenI–taniborbactam complex without a disulfide (PenI-Red-TAN), (**d**) PenI–taniborbactam complex with a disulfide (PenI-Ox-TAN), (**e**) PenI–taniborbactam complex (protonated Glu166) without a disulfide (PenI-Red-TAN-166), and (**f**) PenI–taniborbactam complex (protonated Glu166) with a disulfide (PenI-Ox-TAN-166). The result in a suggests that the contact between Glu166 and Asn170 is well maintained during the simulation. The result in c reveals about 50% maintenance in contact. The results in (b–f) indicate that the interactions are not maintained for most of the simulation time.

**Fig 8:**
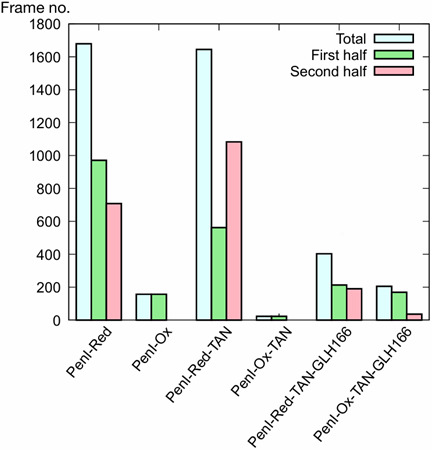
Number of frames where deacylation water (DW) is present during the 200 ns (4,000 frames) simulations. PenI-Red: apo PenI without a disulfide, PenI-Ox: apo PenI with a disulfide, PenI-Red-TAN: PenI–taniborbactam complex without a disulfide, PenI-Ox-TAN: PenI–taniborbactam complex without a disulfide, and GLH166 means protonated Glu166. The light blue bar indicates the number of frames including DW for the total simulation (4,000 frames). The green bar represents the first half of the simulation (2,000 frames). The pink bar represents the second half of the simulation (2,000 frames). The DW in each of the frames was confirmed by the fact that its oxygen atom is less than 3 Å away from Ser70:OG, Glu166:OD1 or OD2, and Asn170:OD1, respectively.

Next, the deacylation water, which was hydrogen-bonded to Glu166 and Asn170, was monitored over the 200 ns simulation (Fig. 7 and 8). The deacylation water was defined as the water located within 3.1 Å of Glu166:OE1 or OE2, 3.1 Å of Asn170:OD1, and 4 Å of Ser70:CA. The simulation of the apo-PenI, where only free thiol groups were present on Cys77 and Cys123 (PenI-Red), showed that the deacylation water was predominantly present, as the distance between Glu166:OE1/2-Asn170:ND2 (OE1 and OE2 of ionized Glu166 are equivalent) was less than 3.0 Å (Fig. 7a). The same distance was maintained 50% of the time in the simulation of the PenI-Red-TAN complex (Fig. 7c), implying that the weakened interactions between Glu166:OE1/2-Asn170:OD2 upon taniborbactam binding might make the deacylation environment unstable. For the other simulation conditions—PenI-Ox (Fig. 7b), PenI-Ox-TAN (Fig. 7d), PenI-Ox-TAN-166 (Fig. 7e), and PenI-Red-TAN-166 (Fig. 7f)—the distance was more than 3 Å for the majority of the time points. Especially in the case of protonated Glu166, OE1, and OE2, position exchange by rotating 180 degrees around the Cγ-Cδ bond was not expected to happen (Fig. 6c). From this observation, two things can be inferred: (i) the deacylated water cannot be stably retained in protonated Glu166, and (ii) the formation of the disulfide bond is involved in the stability of the deacylated water in the active center.

Lastly, Fig. 8 shows how many frames the deacylation water was present in a 200 ns (4,000 frame) trajectory. In the case of PenI-Red and PenI-Red-TAN, the deacylation water was observed for more than 40% of frames (>1,600 frames). However, the deacylation water was present in fewer than 10% (<400 frames) of the entire MD trajectory for PenI-Ox-TAN.

## DISCUSSION

Developing new treatment options effective against *B. pseudomallei* is a public health priority. The heterologous expression of PenI and variants in *E. coli* from a high-copy-number plasmid may simulate induced protein expression levels in the *B. pseudomallei* pathogen, acting as a proxy model to test novel compounds. In *E. coli*, the high level of PenI activity was readily inhibited by both avibactam and taniborbactam. Expression of the PenI variants present in CAZ-resistant strains clearly demonstrated elevated CAZ MIC values even in the presence of avibactam. In contrast, the MIC value for FEP did not differ between wild-type PenI and its CAZ-resistant variants, C69Y and P167S; taniborbactam was able to decrease the FEP MIC value to the vector control level in both cases. Moreover, the 8-fold decrease of the FEP MIC value of the *B. pseudomallei* strain Bp82 confirms that cefepime–taniborbactam is a promising agent for the treatment of *B. pseudomallei* isolates, including CAZ-resistant strains.

Despite elevated MIC of CAZ and FEP against *E. coli* strains expressing PenI C69Y, this variant exhibited reduced hydrolytic activity against these antibiotics *in vitro* compared to wild-type PenI. However, compared to wild-type PenI, the C69Y variant had significantly higher affinity for CAZ and FEP. Similar observations have been noted for the C69F variant of PenI, which is associated with an elevated CAZ MIC (52). Along with increased CAZ affinity, it was proposed that this variant can exist in two isoforms in the bacterial cell, including one that rapidly hydrolyzes CAZ (52). It is possible that such mechanisms are also operative for PenI C69Y. Moreover, inhibition of nitrocefin hydrolysis by C69Y occurred at lower concentrations of CAZ relative to FEP, suggesting that C69Y has a higher affinity for CAZ relative to FEP (Fig. 3). The biochemical and structural characterization of the C69Y variant of *B. thailandensis* PenL, a close homolog of PenI, shows that mutations in the non-catalytic region can induce significant dislocation of β3-β4 strands, as well as conformational changes in critical residues associated with substrate binding and dynamic fluctuation of the Ω-loop and β3-β4 elements (53). These structural changes enlarge the active site and increase its regional flexibility, making it able to accommodate third-generation cephalosporin antibiotics, such as CAZ (53). These observations are reminiscent of interactions between CAZ and KPC variants that drive CAZ-AVI resistance (54, 55) and may partially account for the further elevation of CAZ MIC by the C69Y variant relative to PenI, while the FEP MIC remained essentially unchanged in the presence of PenI or the C69Y variant.

The PenI–taniborbactam crystal structure was very similar to that of KPC-2 bound to taniborbactam (49). Differences included that (i) the terminal 2-aminoethyl moiety of taniborbactam could not be resolved and (ii) the orientation of Trp105 in KPC-2 was different, leading to alternative conformations of taniborbactam in the KPC-2 active site. A unique observation was revealed in the PenI–taniborbactam structure with the presence as well as the absence of the disulfide bond between Cys77 and Cys123 (1:1 ratio) upon taniborbactam binding. As the disulfide bond was not observed in the apo-PenI structure, we hypothesize that the binding of taniborbactam induces a conformational change that slightly shortens the distance between the Cys residues, allowing the formation of the disulfide bond. However, the identity of the electron acceptor required for the formation of the covalent bond between the two thiol side chains is unknown. Furthermore, since the periplasm of Gram-negative bacteria is an oxidizing environment (56, 57), we cannot rule out the possibility that a disulfide bond may form during the inhibition of PenI by taniborbactam. This could potentially increase the residence time of the inhibitor (*t*_1/2_) and enhance its inhibitory effect. However, we believe that the involvement of the Dsb system is less likely. The Dsb system is responsible for generating disulfide bonds in unfolded or partially folded translocated proteins when the cysteines are accessible and the protein scaffold is still flexible (58). In contrast, taniborbactam binds to the fully folded and active form of PenI, where both Cys77 and Cys123 are buried and not accessible to DsbA.

Some class A β-lactamases have either a Cys77–Cys123 or a Cys69–Cys238 disulfide bond. TEM and SHV β-lactamases possess a Cys77–Cys123 disulfide bond, whereas class A carbapenemases, such as SME, GES, and KPC, have a Cys69–Cys238 disulfide bond (59). The PenA class A carbapenemase from *Burkholderia multivorans* does not have either disulfide bond. In general, disulfide bonds are formed between cysteine residues that are sterically close to each other. However, in the PenI β-lactamase crystal structure, although the side chains of Cys77 and Cys123 were close enough to form a disulfide bond, no evidence of a disulfide bond between them was observed in the apo-enzyme, and the side chains remained as free thiols (14). This structural feature is unusual in class A β-lactamases. In the structure of the PenI–taniborbactam complex determined in this study, about 50% of the cysteines formed a disulfide bond. The electron density map as shown in Fig. 5 is reminiscent of a modification occurring after radiation damage (60), but only the PenI–taniborbactam complex, not the apo-PenI structure, has evidence for disulfide formation, suggesting that radiation damage during X-ray data collection is not playing a role.

We then investigated the effect of the formation of the disulfide bond on the overall structure and movement of the main chain amino acid residues by MD simulations. Results suggested that the formation of the disulfide bond upon taniborbactam binding did not affect the main chain amino acids including the state of Glu166. In contrast, the formation of the disulfide bond in PenI decreased the stability of deacylation water. The 200 ns MD simulation from the forced disulfide bond model showed that the hydrogen bond network of Glu166–deacylation water–Asn170 was unstable, and the probability of the presence of deacylation water at the bottom of the active site pocket decreased. Thus, it is likely that the binding of taniborbactam by PenI led to the formation of the disulfide bond between Cys77 and Cys123, which would increase the stability of the PenI–taniborbactam complex and the residence time for the presence of taniborbactam in the complex.

This simulation is the first report of an example where the Cys77–Cys123 disulfide bond outside the active center pocket affected the Ω-loop (i.e., comprised of amino acid residues R164–D179 in class A β-lactamases) and the deacylation water. Previous studies have shown that the removal of the Cys77–Cys123 disulfide bond in TEM-1 resulted in decreased protein stability without the loss of activity (61). In Toho-1, where the Cys77–Cys123 disulfide bond is not present, artificial introduction of the disulfide bond improved protein stability without much effect on activity (62). These results are different from the case of PenI, as taniborbactam artificially induced a disulfide bond and decreased the probability of the existence of the deacylation water, which was thus observed only in the complex and not the apo-enzyme in both the X-ray structure and MD simulation. However, there are still questions as to whether this is a PenI-only phenomenon and why the disulfide bond is not formed in apo-enzyme. To answer these questions, more research is needed.

In conclusion, we demonstrated that PenI, the main mechanism of resistance to β-lactams in the pathogen *B. pseudomallei*, is effectively overcome by potent inhibition by taniborbactam. This is shown in the significant decrease in the FEP MIC values for *E. coli* expressing PenI and its CAZ resistance-conferring variants. Moreover, structural and molecular models suggest that taniborbactam induces the formation of the disulfide bond between Cys77 and Cys123, which destabilizes the diacylation water, strengthening the taniborbactam–PenI complex. These results support further development of cefepime–taniborbactam as a promising alternative treatment of infections by *B. pseudomallei,* including CAZ-resistant strains.

## MATERIALS AND METHODS

### Antibiotics

Taniborbactam (VNRX-5133) and avibactam were provided by Venatorx. Cefepime was obtained from USP and ceftazidime was obtained from Sigma.

### Bacterial strains

For susceptibility testing, engineered *Escherichia coli* DH10B carrying the control plasmid (pTU646) and plasmids expressing PenI-2 (alternative name: BPS-1) or PenI-2 variants were constructed by Twist BioScience, as described previously (34). DNA and amino acid sequences were obtained from the Beta-Lactamase Database (http://www.bldb.eu) (63). Each PenI-2 protein was expressed as an N-terminal fusion with the TorA signal sequence (Tss), which is required for secretion of folded proteins into the periplasm via the twin-arginine translocation system in *E. coli* (45). The Tss was used because PenI is secreted via the cognate system in *B. pseudomallei* (64). The control plasmid (pTU646) is identical to pTU501 (pBR origin, *cat* chloramphenicol-resistant gene, GenBank: MN307371.1) with the addition of a sequence (300 base pairs) that included the coding frame of the Tss.

*Burkholderia pseudomallei* Bp82, a BSL-2 Δ*purM* adenine auxotroph strain (generously provided by Henry S. Heine, University of Florida College of Medicine), was cultured on Mueller-Hinton agar (MHA) and in CAMHB supplemented with 0.6 mM adenine (TCI America A015025G).

### Enzyme expression and purification

The sequence encoding mature PenI-2 (wild-type/WT) or the C69Y variant was cloned into plasmid pET29b to facilitate IMAC purification via a C-terminal hexahistidine tag (Twist Bioscience). Expression of PenI-2 and its variant was driven from an inducible T7 promoter contained in the pET29b backbone. For crystallography, the *bla*_PenI_ gene originally cloned from chromosome 2 of *B. pseudomallei* 1026b (GenBank accession number CP002834.1), minus the first 90 nucleotides, which encode its signal peptide, was previously cloned into the pET24a(+) plasmid using the *Nde*I and *Bam*HI restriction sites, leading to the addition of an ATG (methionine) codon at the 5ʹ end of the gene, and expressed in *E. coli* Origami 2(DE3) cells for protein purification (14). The PenI protein coded in the 1026b strain is PenI-5. PenI-5 has an Ala147Thr substitution relative to PenI-2 which was used for the MIC and biochemical assays.

For kinetics assays, proteins (PenI-2 and its C69Y variant) were expressed in *E. coli* BL21 (DE3) transformed with pET29b expression plasmids following induction with isopropyl β-d-1-thiogalactopyranoside (IPTG) induction for 18 h at 18°C. Cells were harvested, lysed by sonication, and centrifuged at 23,426 × *g* for 30 min. The clarified supernatant was applied to Ni^2+^–nitrilotriacetic acid (NiNTA) resin pre-equilibrated in a buffer comprised of 20 mM sodium phosphate (pH 7.4), 20 mM imidazole, and 10% glycerol. The resin was washed with five column volumes of the above buffer, and the target protein was eluted with stepwise increasing concentrations of imidazole. Fractions were analyzed by sodium dodecyl sulfate–polyacrylamide gel electrophoresis (SDS-PAGE) and assessed for nitrocefinase activity. Fractions containing active β-lactamase were pooled, concentrated, and buffer-exchanged into 20 mM sodium phosphate (pH 7.4) with 5% glycerol prior to storage at −20°C.

For crystallography, *E. coli* Origami 2(DE3) cells carrying pET24a(+) *bla*_penI-5_ were used for protein expression and purification, as previously described (14). Briefly, cells were grown in Super Optimal broth, and then, isopropyl-β-d-1-thiogalactopyranoside was added to induce expression. Cells were pelleted and frozen at −80°C. Subsequently, the cells were lysed, and the PenI-5 β-lactamase was purified via preparative isoelectric focusing electrophoresis and verified by electrospray ionization mass spectrometry, as previously described (14). The final molecular weight of PenI-5 based on electrospray ionization mass spectrometry was 28,345 ± 5 Da with 266 amino acids (theoretical molecular weight: 28,350.18 Da), and the protein was stored in 10 mM phosphate-buffered saline, pH 7.4 with 20% glycerol at a final concentration of 1 mg/mL. The numbering system for class A β-lactamases established by Ambler et al. was the convention used to refer to specific amino acids (65).

### Susceptibility testing

Isogenic *E. coli* strains were cultured in cation-adjusted Mueller-Hinton broth (CAMHB) supplemented with 10 µg/mL chloramphenicol to maintain the plasmid. Broth microdilution minimum inhibitory concentration assays (MIC) were performed in CAMHB according to CLSI guidelines (66) with ceftazidime (CAZ) and cefepime (FEP) tested alone or in combination with avibactam or taniborbactam, respectively. The β-lactamase inhibitors, taniborbactam and avibactam, were fixed at a concentration of 4 µg/mL. To ensure reliability of MIC results, two quality control strains were utilized, *K. pneumoniae* ATCC 700603 and *E. coli* NCTC-13353 (Table S2). MIC testing using the *B. pseudomallei* strain Bp82 was performed as described with the addition of 0.6 mM adenine to the CAMHB. The MIC value was defined as the lowest concentration of antibiotic with no visible growth. Modal MIC values of three replicates were determined.

### Quantification of β-lactamase activity in the cell lysate

Isogenic *E. coli* strains were grown to log phase at an OD_600_ of ~0.5 in CAMHB supplemented with 10 µg/mL chloramphenicol. Cells from 1 mL culture were harvested by centrifugation. The cell pellet was freeze-thawed, resuspended in 100 µL BugBuster HT Protein Extraction Reagent (Millipore Sigma), and incubated at room temperature for 15 min with shaking, followed by centrifugation at 21,100 × *g* for 20 min. The supernatant (cell lysate) was taken and used in the Bradford assay to measure the protein concentration and in the β-lactamase activity assay. In the β-lactamase activity assay (total reaction volume: 150 µL), cell lysate (5 µL) was incubated with 100 µM nitrocefin in 1 × phosphate-buffered saline (pH 7.4) containing 0.1 mg/mL bovine serum albumin (BSA), and absorbance at 486 nm was measured for 30 min. β-Lactamase-specific activity (nanomoles of nitrocefin hydrolyzed per minute per milligram of protein) was determined using the nitrocefin extinction coefficient of 17,400 M^−1^cm^−1^.

### Steady-state kinetics

Kinetic parameters for substrate hydrolysis by PenI-2 were determined under steady-state conditions. Reactions were performed in triplicate in sodium phosphate buffer (pH 7.4) with PenI enzyme at a final concentration of 11 nM (200 nM for CAZ reactions) and with increasing concentrations of substrate: nitrocefin, FEP, CAZ, or MEM. Hydrolysis was continuously monitored by measuring changes in absorbance at 486 nm for nitrocefin, at 260 nm for CAZ and FEP, or 298 nm for MEM. Initial rates of hydrolysis were plotted as a function of substrate concentration, and the data were fit to the Michaelis–Menten equation (equation 1) to derive *K*_M_ and *k*_cat_, where *v* is the initial velocity, [E]_0_ is the initial enzyme concentration, [S] is the substrate concentration, *k*_cat_ is the catalytic rate constant, and *K*_M_ is the Michaelis constant:


(1) $$v=\frac{\left[E{]}_{0}\times k_{cat}\times [S\right]}{K_{M}+[S]}$$

Unlike nitrocefin, the initial rate of CAZ hydrolysis by PenI, and of CAZ or FEP hydrolysis by the C69Y variant, did not approach saturation with increasing concentrations of these antibiotics. Consequently, the corresponding kinetic parameters for hydrolysis could not be determined. Therefore, the progress of FEP and CAZ hydrolysis by equivalent concentrations of PenI and the C69Y variant was compared.

Half-maximal inhibition (IC_50_) values were determined by pre-incubating increasing concentrations of β-lactamase inhibitors with PenI for 15 min. Nitrocefin was added, and the increase in absorbance at 486 nm was monitored for up to 15 min. The IC_50_ was determined by plotting the fractional activity versus the inhibitor concentration and fitting equation 2 to the data, where *y* is the fractional activity at a given inhibitor concentration, *y*_min_ is the fractional activity when the enzyme is completely inactivated, *y*_max_ is the maximum (uninhibited) fractional activity, *n* is the Hill coefficient, and *x* is the inhibitor concentration:


(2) $$y=y_{\min}+\left[y_{\max}-y_{\min}\right]/\left[1+{\left(\frac{x}{{IC}_{50}}\right)}^{n}\right]$$

To determine the *k*_2_/*K* for β-lactamase inhibitors with PenI, increasing concentrations of test compounds were mixed with nitrocefin, and the reactions initiated by addition of enzyme. The increase in absorbance at 486 nm was monitored continuously for up to 30 min. The full set of progress curves for all inhibitor concentrations was analyzed by numerical integration using the Kintek Global Kinetic Explorer (Kintek Corp. Snowshoe PA) and fit to the kinetic model below to obtain kinetic parameters as previously described (67).


Scheme 1 $$E+S\overset{k1}{\underset{k_{-1}}{⇄}}ES\overset{k2}{\underset{k_{-2}}{⇄}}E+P$$

Scheme 2 $$E+I\overset{k3}{\underset{k_{-3}}{⇄}}EI$$

In the model, the concentration of enzyme, nitrocefin, the enzyme–nitrocefin complex, the nitrocefin hydrolysis product, inhibitor concentration, and enzyme–inhibitor complex are denoted by E, S, ES, P, I, and EI, respectively (Scheme 1). The parameter *k*_3_ corresponds to the second-order rate constant *k*_2_/*K* (Scheme 2) (67).

The dissociation rate (*k*_off_) was determined by the jump dilution method as previously described using nitrocefin as the substrate (34). Enzyme–inhibitor (1:2 ratio) complexes were diluted 4,000-fold overall, and the recovery of nitrocefin hydrolysis activity was measured continuously by the increase in absorbance at 486 nm. Reaction progress curves were fit to a single exponential function (equation 3), where *y* is the absorbance at each time point, *A*_*i*_ is the background absorbance, *y*_*i*_ is the initial absorbance, *t* is time, and *k is* the off rate.


(3) $$y=A_{i}+\left[y_{i}\;e^{kt}\right]$$

The half-life or t_1/2_ was determined using equation 4.


(4) $$t_{1/2}=\frac{\ln(2)}{k}$$

The apparent inhibitory constant *K*_i_ (*K*_i app_) of each BLI for PenI and the C69Y variant was determined in a direct competition assay that measured the initial rate of nitrocefin hydrolysis in the presence of increasing concentrations of inhibitor. The observed *K*_i app_ (*K_i_*_app observed_) was first derived from the linear plot of the inverse initial velocity vs. inhibitor concentration, by dividing the value for the y-intercept of the line by the slope. The data were then corrected for nitrocefin affinity using equation 5.


(5) $$K_{i\;app}=K_{i\;app\;observed}/[1+\frac{\left[S\right]}{K_{M}(nitrocefin)}]$$

### Crystallization and data refinement

From the stock solution (1 mg/mL), PenI-5 was concentrated and buffer-exchanged into crystallization buffer to reach 7.5 mg/mL PenI protein (12.5% PEG8K, 0.05 M ammonium sulfate, 0.05 M HEPES, pH 7.5). PenI was crystallized by the vapor diffusion method using a 250 µL reservoir (25% PEG8K, 0.1 M ammonium sulfate, 0.1 M HEPES, pH 7.5) with a 4 µL hanging drop (7.5 mg/mL PenI protein, 12.5% PEG8K, 0.05 M ammonium sulfate, 0.05 M HEPES, pH 7.5). Needle-shaped crystals appeared in 1 week, reaching sizes of 0.4–0.6 × 0.02–0.05 mm. Before data collection, a pre-grown crystal was soaked at room temperature for 30 min in the holding solution (30% PEG8K, 0.1 M ammonium sulfate, 0.1 M HEPES, pH 7.5) containing 1 mM taniborbactam (dissolved from powder directly into the holding solution). The crystals were cryoprotected by dipping them into a reservoir solution containing 20% glycerol, flash-cooled, and kept at 100 K with a nitrogen gas stream. The 1.0° oscillation images were collected on an Eiger X16M detector (DECTRIS AG, Baden, Switzerland) with synchrotron radiation at beamline BL-17A of the Photon Factory, Tsukuba, Japan. Only one crystal was used. The XDS programs were used to reduce and scale X-ray intensities (Table S1) (68). Molecular replacement using the PenI structure (PDB 3W4P) as a search model and model refinement was done using the PHENIX program (69). Coot was used for manual model fitting (70). PDB code assigned for the PenI–taniborbactam structure is 9JSE.

### Molecular dynamics

The initial coordinates of the atoms in the apo-PenI-5 and PenI-5:taniborbactam complex were from respective crystal structures (PDB:3W4P and this study, respectively). The crystallographic waters, except for the deacylation and oxyanion waters, were removed, and then, water molecules were generated around the inhibitor and enzyme by the 3D-RISM atom placement algorithm using Amber20 program (71, 72). The PenI molecule was placed in a rectangular box filled with TIP3P water molecules. The model also contained sodium and chloride ions to neutralize the model system. The final model size was ca. 95 Å × 90 Å × 99 Å, and the total number of atoms was about 48,800 for six models. The ff14SB force field was applied to the enzyme (73). Minimization, heating, and pre-equilibration were carried out using the sander module of Amber20. A production run of MD simulation was carried out using the pmemd module (74). The calculation procedure used was similar to previous works (75–77). The production runs of MD simulation were carried out for 200 ns. The cutoff distance for the electrostatic and van der Waals energy terms was set to 12.0 Å. The Particle Mesh Ewald method was applied to calculate the long-distance electrostatic force. The integration time step was 1 fs. The ptraj module was utilized to obtain the snapshot structures from the simulation trajectory, the root-mean-square deviation (RMSD), and the *B*-factor. In the calculation of the *B*-factor, only main chain atoms (N, CA, and C) were considered.

### Biosafety statement

*B. pseudomallei* Bp82 is a Δ*purM* adenine auxotroph strain, which has been proven to be completely avirulent in hypersusceptible animal models, including BALB/c mice and the Syrian hamster challenge model. Moreover, Bp82 also failed to cause mortality in immune-deficient mice and to replicate *in vivo* or disseminate following intranasal challenge (47). Because of this complete attenuation, Bp82 is safe for use under BSL-2 laboratory conditions and has been excluded from the requirements of 9 CFR Part 121 and 42 CFR Part 73 of the CDC and the United States Department of Agriculture (USDA) Select Agent Status list (78).

## SUPPLEMENTAL MATERIAL

Supplemental material aac.00787-25-s0001.docx Tables S1 and S2; Fig. S1.

ASM does not own the copyrights to Supplemental Material that may be linked to, or accessed through, an article. The authors have granted ASM a non-exclusive, world-wide license to publish the Supplemental Material files. Please contact the corresponding author directly for reuse.
