# Supplementary material for: Burkholderia pseudomallei PenI β-lactamase and variants are potently inhibited by taniborbactam
Source: Antimicrob Agents Chemother. 2025 Sep 12;69(10):e00787-25. doi: 10.1128/aac.00787-25 (PMC12486822; doi:10.1128/aac.00787-25)
Supplement: Supplemental material — Tables S1 and S2; Fig. S1. [file aac.00787-25-s0001.docx]

**Supplementary information**

***Burkholderia pseudomallei* PenI β-lactamase and Variants Are Potently Inhibited by Taniborbactam**

Maria F. Mojica^1,2,3^, Scott A. Becka^2^, Mitchell Edwards^4^, Cullen Myers^4*^, Kyoko Uehara^4^, Tsuyoshi Uehara^4^, Tyuji Hoshino^5^, Elise T. Zeiser^2^, Cassandra L. Chatwin^4^, David A. Six^4^, Robert A. Bonomo^1,3,6,7,8,9,10^ Krisztina M. Papp-Wallace^7,8†^ Michiyoshi Nukaga^11†^

^1^Department of Molecular Biology and Microbiology, Case Western Reserve University School of Medicine, Cleveland, Ohio, USA; ^2^Research Service, Veterans Affairs Northeast Ohio Healthcare System, Cleveland, Ohio, USA; ^3^CASE-VA Center for Antimicrobial Resistance and Epidemiology, Cleveland, Ohio, USA; ^4^Venatorx Pharmaceuticals, Inc., Malvern, Pennsylvania, USA; ^5^Graduate School of Pharmaceutical Sciences, Chiba University, Chuo-ku, Chiba, Japan; ^6^ Geriatric Research, Education and Clinical Center, Louis Stokes Cleveland VA Medical Center, Cleveland Ohio, USA, ^7^Department of Medicine, Case Western Reserve University School of Medicine, Cleveland, Ohio, USA; ^8^Department of Biochemistry, Case Western Reserve University School of Medicine, Cleveland, Ohio, USA; ^9^Department of Pharmacology, Case Western Reserve University School of Medicine, Cleveland, Ohio, USA; ^10^Department of Proteomics and Bioinformatics, Case Western Reserve University School of Medicine, Cleveland, Ohio, USA; ^11^Department of Pharmaceutical Sciences, Josai International University, Togane City, Chiba, Japan

Table S1: Data collection and refinement statistics

| **PenI-taniborbactam complex*** | |
| --- | --- |
| **Data collection** | |
| **Beamline (Detector)** | PF BL-17A (Dectris Eiger X16M) |
| **Wavelength (Å)** | 0.98 |
| **Resolution range (Å)** | 32.6 - 1.25 (1.295 - 1.25) |
| **Space group** | *P*2_1_ |
| **Molecules/ASU** | 1 |
| **Cell dimensions: *a, b, c* (Å), a, b, g (°)** | 41.20, 52.12, 50.44, 90.00, 92.55, 90.00 |
| **Total reflections** | 197656 (18720) |
| **Unique reflections** | 56254 (5285) |
| **Redundancy** | 3.5 (3.5) |
| **Completeness (%)** | 95.30 (90.56) |
| **Mean *I/s(I)*** | 16.02 (3.12) |
| ***R*_merge_** | 0.04258 (0.3798) |
| **CC_½_ (%)** | 0.999 (0.867) |
| **Refinement** | |
| **No. reflections** | 56253 (5285) |
| ***R*_work_/*R*_free_** | 0.1252(0.191) / 0.151 (0.215) |
| **Protein residues** | 264 |
| **RMS bond length(Å)**  **Bond angles (°)** | 0.009  1.11 |
| **Ramachandran favored (%)**  **Allowed**  **Outliers** | 99.24  0.76  0.00 |
| **Average *B*-factor**  **Total**  **Macromolecules**  **Ligands**  **Solvent** | [No., non-H atoms]  13.98 [2348]  12.45 [2055]  15.70 [69]  25.89 [259] |

*For each row, the statistics are shown for the entire data set followed by parenthesis containing the statistics for the highest-resolution shell.

**Table S2. Minimum Inhibitory Concentration values of Quality Control Strains**

| **Strain** | **Strain ID** | **Minimum Inhibitory Concentration (µg/mL)** | | | |
| --- | --- | --- | --- | --- | --- |
|  |  | **CAZ** | **CAZ+AVI*** | **FEP** | **FEP+TAN*** |
| *E. coli* | NCTC 13353 | NA | NA | 64 | 0.25 |
| *K. pneumoniae* | ATCC 700603 | 32 | 0.5 | 0.5 | 0.12 |

*AVI and TAN were fixed at 4 µg/mL; AVI, avibactam; CAZ, ceftazidime; FEP, cefepime; TAN, taniborbactam; NA, not applicable.

**
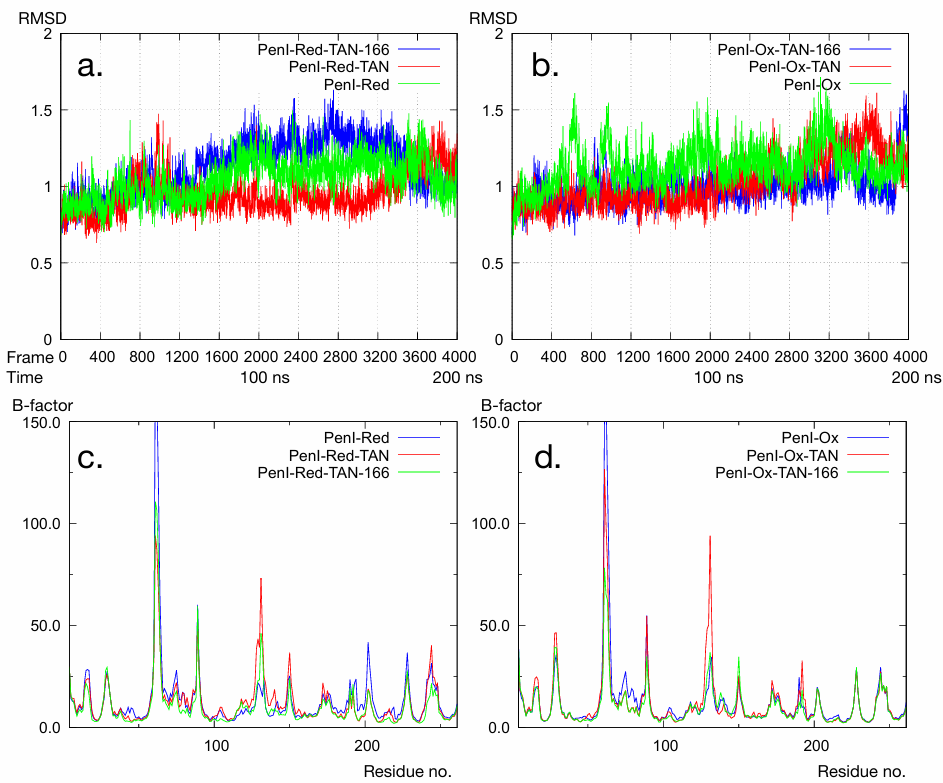
**

**Figure S1** Changes in the RMSD and average *B*-factors for main chain atoms during the 200 ns MD simulations. **a.** Changes in the RMSD for main-chain atoms during the 200 ns simulations relative to the starting structure PenI without a disulfide bond (PenI-Red). **b.** Changes in the RMSD for main-chain atoms during the 200 ns simulations relative to the starting structure PenI with a disulfide bond (PenI-Ox, green). The blue line color indicates: PenI-taniborbactam complex with protonated Glu166 (PenI-Ox-TAN-166) and the red line color indicates: PenI-taniborbactam complex with deprotonated Glu166 (PenI-Ox-TAN). **c.** Average *B*-factors of main-chain atoms of the individual amino acid residues during the 200 ns MD simulations PenI without a disulfide bond (PenI-Red) and **d.** Average *B*-factors of main-chain atoms of the individual amino acid residues during the 200 ns MD simulations PenI with a disulfide bond (PenI-Ox). The color codes are the same as in **a.** and **b.**
